# The Signaling and Pharmacology of the Dopamine D1 Receptor

**DOI:** 10.3389/fncel.2021.806618

**Published:** 2022-01-17

**Authors:** Jace Jones-Tabah, Hanan Mohammad, Emma G. Paulus, Paul B. S. Clarke, Terence E. Hébert

**Affiliations:** Department of Pharmacology and Therapeutics, McGill University, Montreal, QC, Canada

**Keywords:** G protein-coupled receptors, dopamine D1 receptor, intracellular signaling, drug development, biased agonism, Parkinson's Disease, cognitive impairment (CI)

## Abstract

The dopamine D1 receptor (D1R) is a Gα_s/olf_-coupled GPCR that is expressed in the midbrain and forebrain, regulating motor behavior, reward, motivational states, and cognitive processes. Although the D1R was initially identified as a promising drug target almost 40 years ago, the development of clinically useful ligands has until recently been hampered by a lack of suitable candidate molecules. The emergence of new non-catechol D1R agonists, biased agonists, and allosteric modulators has renewed clinical interest in drugs targeting this receptor, specifically for the treatment of motor impairment in Parkinson's Disease, and cognitive impairment in neuropsychiatric disorders. To develop better therapeutics, advances in ligand chemistry must be matched by an expanded understanding of D1R signaling across cell populations in the brain, and in disease states. Depending on the brain region, the D1R couples primarily to either Gα_s_ or Gα_olf_ through which it activates a cAMP/PKA-dependent signaling cascade that can regulate neuronal excitability, stimulate gene expression, and facilitate synaptic plasticity. However, like many GPCRs, the D1R can signal through multiple downstream pathways, and specific signaling signatures may differ between cell types or be altered in disease. To guide development of improved D1R ligands, it is important to understand how signaling unfolds in specific target cells, and how this signaling affects circuit function and behavior. In this review, we provide a summary of D1R-directed signaling in various neuronal populations and describe how specific pathways have been linked to physiological and behavioral outcomes. In addition, we address the current state of D1R drug development, including the pharmacology of newly developed non-catecholamine ligands, and discuss the potential utility of D1R-agonists in Parkinson's Disease and cognitive impairment.

## Introduction to D1R Pharmacology

The neurotransmitter dopamine plays fundamental roles in governing voluntary movement, processing motivational stimuli, and facilitating learning. The importance of dopamine signaling is underscored by its role in substance abuse disorders and in the debilitating symptoms associated with dopaminergic dysfunction. Dysfunctional dopamine signaling in various brain regions is a causal factor in many symptoms of neurodegenerative and neuropsychiatric disorders including Parkinson's Disease (PD), substance abuse, schizophrenia, autism and attention deficit hyperactivity disorder. The diversity of clinical presentation across these conditions highlights the complexity of dopamine functions in the central nervous system (CNS), and hints at the potential power of dopaminergic pharmacotherapy. Indeed, many drugs that modulate the levels or release of dopamine are used to treat the aforementioned disorders. More specifically, drugs that directly target the dopamine D1 receptor (D1R) have been investigated for the treatment of motor impairment in Parkinson's Disease, and cognitive impairment associated with age, neuropsychiatric, and neurodegenerative diseases. However, despite numerous animal and human studies supporting the effectiveness of D1R-targeting pharmacotherapies, no selective D1R ligands have yet been deployed clinically. Here, we will discuss current knowledge of D1R-mediated cellular signaling, and recent developments in D1R pharmacology, focusing on how a deeper understanding of receptor signaling can inform strategies for drug development.

Dopamine receptors are G protein-coupled receptors (GPCRs) and are classified into two families based on preferential G protein coupling: the D1 and D5 receptors (D1-class) which are canonically coupled to Gα_s/olf_, which stimulates the activity of adenylyl cyclases (AC), and the D2, D3 and D4 receptors (D2-class), which are primarily coupled to Gα_i/o_, which inhibits AC activity. For recent reviews describing the physiology and pharmacology across the dopamine receptor family, see the following references (Beaulieu and Gainetdinov, 2011; Beaulieu et al., 2015; Martel and Gatti McArthur, 2020). Here, we will discuss our current understanding of the signaling pathways activated by the D1R. Furthermore, we will describe how these pathways may differ between specific neuronal subtypes, and under specific circumstances. Building on this information we will then discuss the current state of D1R-targeting pharmacotherapies, and how this knowledge of signaling can inform future drug development aimed at specific facets of D1R-dependent signaling. Beyond the canonical description as a Gα_s/olf_-coupled receptors, the signaling of D1-class dopamine receptors is complex, cell-type dependent, and often altered by specific disease processes. Understanding the dynamics of D1R signaling beyond Gα_s/olf_ will be an important step toward defining cell type-specific signaling associated with desirable therapeutic outcomes and ultimately designing better therapeutics.

The D1R is the most abundant dopamine receptor in both the rodent and human brain, and is expressed in many regions including the dorsal striatum (caudate-putamen), ventral striatum (nucleus accumbens), cortex, thalamus, amygdala, and substantia nigra pars reticulata (Mansour et al., 1990; Hall et al., 1994). Dysfunctional D1R signaling is associated with a host of human disorders which includes PD, schizophrenia, Huntington's disease and attention deficit hyperactivity disorder. In the later sections of this article we will primarily describe two conditions in which pharmacotherapies directly targeting the D1R are currently being developed: PD and cognitive impairment. However, the D1R also plays a role in mediating the effects of indirect dopamine agonists, including the dopamine precursor L-DOPA, used in the treatment of Parkinson's Disease, and stimulants like cocaine and amphetamine, which enhance the release or extracellular persistence of dopamine. Experiments conducted in the context of multiple diseases and using a variety of pharmacological manipulations have all contributed to the current understanding of D1R signaling across different neuronal populations.

### The Clinical Potential of Targeting the D1R

D1R agonists have long been considered for clinical use, most notably for the management of motor dysfunction in Parkinson's Disease (PD), and of cognitive impairment associated with age, neurodegenerative or neuropsychiatric disorders. However, despite nearly 40 years of investigation as a therapeutic target, there are currently no centrally acting D1/D5R ligands in clinical use. The later sections of this review will briefly describe preclinical and clinical studies that investigated the therapeutic potential of D1/D5R agonists and allosteric modulators. Further, we will discuss recent progress following the development of non-catecholamine chemical scaffolds, and how our understanding of D1R signaling can inform the development of new D1R ligands.

**Figure 1:**
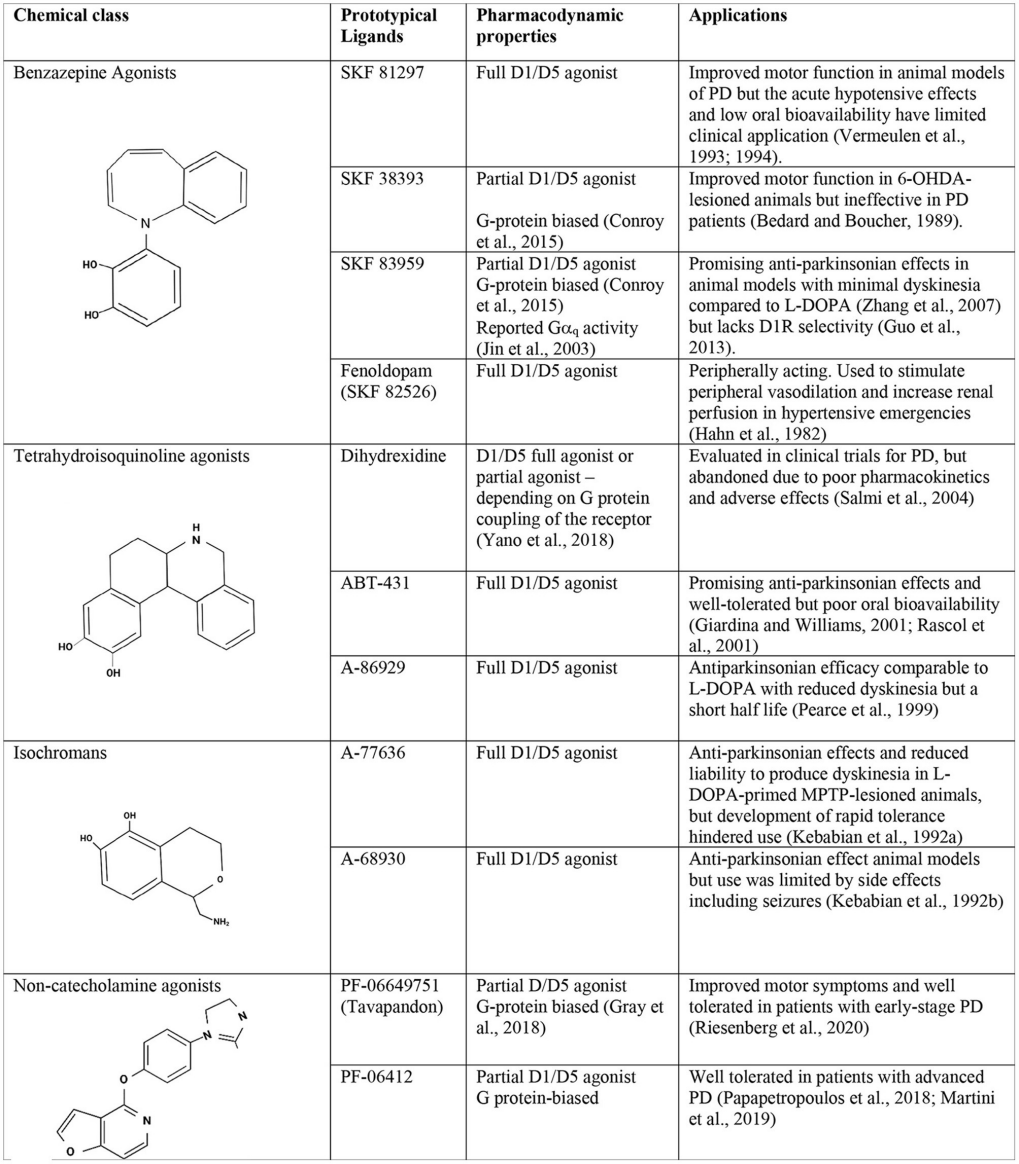
Chemistry and pharmacology of D1R ligands. Chemical structures, pharmacodynamic properties, and tested applications of representative ligands from the principal D1/D5R agonist classes.

Over the years, successive “generations” of D1/D5R agonists have been developed based on varying chemical scaffolds and with a variety of pharmacologic properties (Figure 1). Several have been investigated as potential therapies for PD and cognitive impairment but all failed in clinical or preclinical development due to the chemical properties of the ligands themselves. Specifically, until recently, all selective D1/D5R agonists contained a catechol (i.e., dihydroxyphenyl) group, a chemical moiety which limits oral bioavailability and central nervous system penetration, rendering these compounds susceptible to rapid metabolism (reviewed in Felsing et al., 2019). These chemistry-related limitations precluded their clinical use, and the development of D1/D5R agonists was abandoned for several years. However, D1/D5R ligands are now attracting renewed clinical interest, thanks to the recent development of D1R positive allosteric modulators (PAMs) as well as new agonists based on non-catecholamine scaffolds which provide improved pharmacokinetic properties and greatly reduced off-target effects. Since the development of these molecules, several new clinical studies have been initiated, revisiting the utility of D1/D5R agonists as therapeutics for the treatment of PD and cognitive impairment.

### A New Pharmacology of the D1R

#### Biased Agonism at the D1R

An important emerging concept in recent progress in D1R pharmacology is the property of *biased agonism*, or *functional selectivity*. As will be described throughout the following sections, activation of GPCRs such as the D1R can lead to activation of multiple downstream signaling pathways, separated in both space and time. “Biased” or “functionally selective” ligands are defined by their ability to preferentially promote or inhibit a select subset of the intracellular pathways coupled to a given receptor (reviewed in Reiter et al., 2012). The concept of biased agonism has challenged conventional views of GPCR signaling and has important therapeutic implications. By selectively targeting some pathways over others, biased ligands could in principle provide therapeutic benefit with fewer adverse effects. In addition to offering the ability to selectively target specific signaling pathways, biased ligands also offer the possibility of reducing the development of drug tolerance, by avoiding receptor desensitization.

The most notable example of biased agonism for the D1R is the dissociation of G protein-dependent signaling from β-arrestin recruitment and signaling. Biased agonism has also been described at the D1R in relation to Gα_s_ vs. Gα_olf_ signaling (Yano et al., 2018) and Gα_s_ vs. Gα_q_ (Jin et al., 2003), which will be discussed in section Signal Transduction via the D1R of this review. Recently developed non-catecholamine D1/D5R agonists can be engineered to exhibit a range of functional selectivity profiles, and in particular can dissociate G protein signaling from β-arrestin recruitment (Gray et al., 2018; Wang et al., 2019b; Martini et al., 2019a,b). In a second advance, molecular determinants of both effector coupling and functional selectivity have recently been elucidated from the structures of the D1R in complex with full, partial and biased agonists (Sun et al., 2021; Xiao et al., 2021; Zhuang et al., 2021). The latter findings could facilitate future development of ligands with even greater specificity and functional selectivity through structure-guided design.

Non-catecholamine D1/D5R agonists were first identified in a high-throughput screen, and subsequent optimization led to a series of compounds with favorable oral bioavailability and pharmacokinetic profiles (Gray et al., 2018). Some ligands from this series also displayed G protein bias and were found to produce less receptor desensitization with more sustained *in vivo* activity after repeat dosing, when compared to a typical unbiased agonist (Gray et al., 2018). Subsequent efforts to further optimize the structure of these compounds has led to an array of available agonists with a range of pharmacological properties (Davoren et al., 2018; Wang et al., 2019b; Martini et al., 2019a,b). In mouse models, one such agonist was found to have anti-Parkinsonian effects that were sustained for significantly longer than a typical catecholamine agonist, owing both to reduced receptor desensitization and to a more favorable pharmacokinetic profile (Martini et al., 2019b). Such developments have stimulated renewed interest in the clinical use of D1/D5R agonists.

#### Positive Allosteric Modulators

Positive allosteric modulators (PAMs) are ligands which bind at sites distinct from the orthosteric ligand binding site of a receptor. PAMs do not directly activate signaling, but instead can modulate the affinity or efficacy of orthosteric ligands by modifying receptor conformation or structural transitions. Hence, PAMs can promote D1R activity by either enhancing signaling mediated by endogenous ligands or altering the coupling of the receptor to downstream effectors. In preclinical studies, recently developed D1R PAMs have shown promise in the treatment of cognitive impairment (Bruns et al., 2018; Hao et al., 2019; Svensson et al., 2019), and one such compound (LY3154207, branded as Mevidalen), has progressed into clinical development, where it has been found to be safe and well-tolerated (Wilbraham et al., 2021a,b). Mevidalen improved motor function in patients with Lewy Body Dementia when given in addition to standard dopaminergic therapy (Biglan et al., 2021), and initial findings also hint at a possible alleviation of motor symptoms in Parkinson's Disease patients (Wilbraham et al., 2021b). Mevidalen was also reported to increase wakefulness in both sleep-deprived mice and human subjects (McCarthy et al., 2021).

In addition to these promising clinical findings, D1R PAMs as a class have an additional advantage: since they bind outside the D1R orthosteric site, they can be developed to spare the D5R (Svensson et al., 2017). Such selectivity has so far been unattainable with traditional agonists and antagonists, and in addition to the strong possibility of generating receptor-specific ligands with closely homologous orthosteric sites, this will likely be an invaluable research tool for understanding the relative contributions of such receptors to various functions *in vivo*.

### Pharmacological Selectivity at D1-Class Receptors

An important caveat when considering the signaling and pharmacology of the D1R is the potential contribution of the highly homologous D5R. Due to the high degree of sequence homology (Sunahara et al., 1991) and presumed structural similarity, the two D1-class receptors have generally been considered pharmacologically indistinguishable. Effectively, this means that no currently available ligands display substantial selectivity for either receptor (reviewed in Giorgioni et al., 2008; Bueschbell et al., 2019). Compared to the D1R, the D5R has a more restricted expression profile in the CNS, but is expressed in brain regions involved in mediating the effects of D1R stimulation, including the striatum and prefrontal cortex (Meador-Woodruff et al., 1992; Ciliax et al., 2000; Khan et al., 2000; Rivera et al., 2002). This overlap in regional expression and a lack of selective pharmacological tools has made it challenging to define the specific signaling and physiological functions mediated by each receptor. Moreover, findings from knockout mice have implicated the D5R in processes that may overlap with those regulated by the D1R, including the stimulant effects of cocaine and D1/D5 agonists (Holmes et al., 2001; Elliot et al., 2003; O'Sullivan et al., 2005), spontaneous jaw movements (Tomiyama et al., 2006), dopaminergic induction of seizures (O'Sullivan et al., 2008), regulation of hippocampal spatial memory (Moraga-Amaro et al., 2016), and regulation of working memory and specific signaling pathways in the prefrontal cortex (Perreault et al., 2013; Carr et al., 2017). In contrast, examples of events found to be exclusively mediated by the D5R include regulation of cholinergic striatal interneurons during L-DOPA induced dyskinesia (Castello et al., 2020), and modulation of hippocampal acetylcholine release (Laplante et al., 2004). Although the focus of this review will be the D1R, the inability in many cases to rule out contributions of the D5R is an important caveat to many of the *in vivo* studies described here.

## Signal Transduction Via the D1R

### Receptor-Proximal Signal Transduction: G Proteins and β-Arrestins

**Figure 2:**
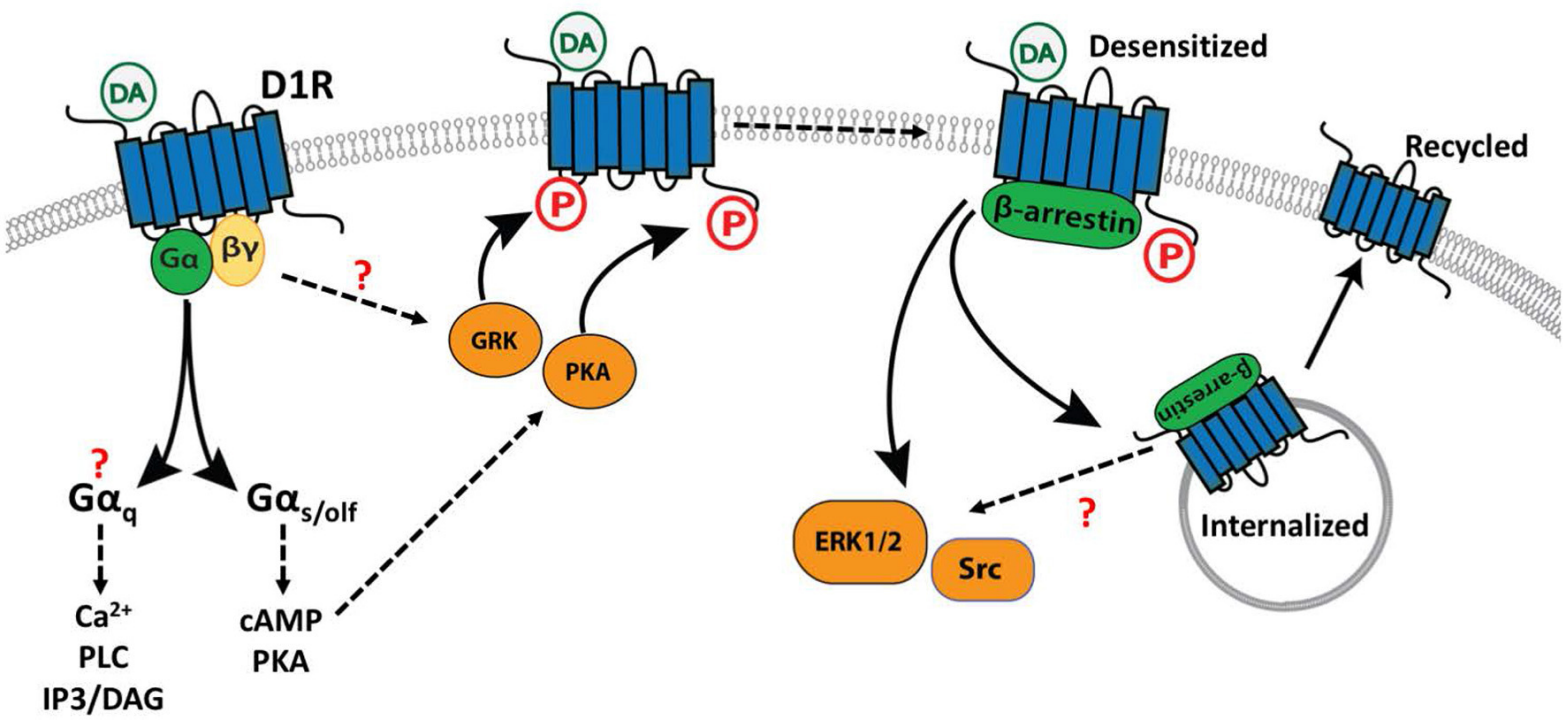
Effector coupling of the D1R. Shown are the main interactions of the D1R with three sets of downstream effectors/modulators. Regulatory interactions that have not been fully elucidated, or for which contradictory evidence exists, are indicated with a “?”. Binding of a ligand such as dopamine (DA) activates G protein-dependent **(Left)** signaling through multiple effectors. Desensitization of the receptor **(Center)** is mediated by phosphorylation (P) of the C terminal tail and intracellular loops by GRKs and PKA. β-arrestin binding to the phosphorylated receptor **(Right)** mediates desensitization, internalization, and potentially additional intracellular signaling events generated through the activation of ERK1/2 and Src protein kinases.

As introduced above, the D1R is canonically coupled to Gα_s/olf_ but may also transduce signals through alternative mechanisms including coupling to Gα_q_, or through recruitment of β-arrestin isoforms (Figure 2). Here, we describe the mechanisms of D1R-mediated signal transduction, and the cellular circumstances under which they occur.

#### Coupling to Gα_s/olf_ and cAMP Signaling

The D1R couples principally to the Gα_s_ family of G proteins, including Gα_s_ and Gα_olf_. These G proteins display different anatomical distributions, with Gα_olf_ predominating in the striatum, and Gα_s_ in regions that include the cortex and hippocampus (Hervé et al., 1993; Zhuang et al., 2000; Hervé, 2011). Gα_s_ and Gα_olf_ proteins are structurally and functionally similar; both stimulate adenylyl cyclase (AC) activity, leading to increased production of the second messenger cyclic adenosine monophosphate (cAMP), which can then activate downstream effectors including protein kinase A (PKA) and *Exchange Protein Activated by cAMP* (EPAC). Nevertheless, Gα_s_ and Gα_olf_ differentially affect D1R pharmacology. For example, dihydrexidine, a ligand initially defined as a D1/D5R full agonist, behaves as such only at Gα_s_-coupled D1Rs and instead acts as a partial agonist at Gα_olf_-coupled D1Rs (Yano et al., 2018). Such functional differences, combined with the different anatomical patterns of expression, means that D1R pharmacology may be region-specific, the consequences of which have only recently begun to be studied. For example, in the striatum Gα_olf_ predominates and expression levels of Gα_olf_ represent a critical determinant of D1R signaling output, as mice that are heterozygous for Gα_olf_ knockout (*Gnal*+*/*) display blunted behavioral and biochemical responses to dopaminergic manipulation (Hervé et al., 2001; Corvol et al., 2007). Furthermore, even within the striatum, Gα_olf_ expression levels vary between compartments, with higher expression being observed in striosomes compared to striatal matrix (Sako et al., 2010), a pattern disrupted in animal models of Parkinson's disease (Ruiz-DeDiego et al., 2015b).

#### Coupling to Gα_q_, PLC, and Intracellular Ca^2+^ Release

Several lines of evidence have suggested that the D1R can couple to Gα_q_, but the physiological circumstances under which such coupling occurs remain controversial. Dating back to the 1980's, it was observed that D1/D5R agonists could stimulate phospholipase C (PLC), a canonical downstream effector of Gα_q_, in a manner that appeared independent of AC stimulation (Felder et al., 1989). Some D1/D5R agonists, notably those of the benzazepine family, stimulate nominally Gα_q_-dependent signaling events in a variety of cellular contexts, but it remains uncertain whether these effects are mediated by direct D1R-dependent activation of Gα_q_ (reviewed in Lee et al., 2014). Thus, while at least two independent groups have shown that in cultured cells D1R is capable of coupling directly to Gα_q_ (Inoue et al., 2019; Okashah et al., 2019), alternative explanations for apparent Gα_q_ signaling *in vivo* have also been offered. One possibility is that benzazepine-based D1/D5R agonists may have off-target effects at high concentrations (reviewed in Lee et al., 2014). Another is that Ca^2+^ mobilization downstream of D1R (and PLC) may be Gβγ-dependent (Chun et al., 2013). A third possibility is that the activation of Gα_q_, PLC or calcium mobilization attributed to the D1R could in some cases be mediated by the D5 receptor. This potential explanation is supported by two findings: (1) the D5R (but not D1R) stimulated calcium mobilization when expressed in HEK 293 cells (So et al., 2009), and (2) D5R knockout, but not D1R knockout prevented D1/D5R agonists from stimulating IP_3_ production in mouse brain membranes and slices (Friedman et al., 1997; Sahu et al., 2009).

An alternate hypothesis relating D1R to Gα_q_ is that while this receptor does not couple to Gα_q_ on its own, it does so through the formation of a D1R-D2R heterodimer (Lee et al., 2004; Rashid et al., 2007; Perreault et al., 2011). Evidence supports the co-expression and proximal co-localization of the D1 and D2 receptors in multiple brain regions in both rodents and primates (Perreault et al., 2010, 2011; Hasbi et al., 2020) and a D1-D2 heterodimer has been proposed to have functional significance in animal models of Parkinson's Disease (Rico et al., 2017), depression (Shen et al., 2015), anxiety (Shen et al., 2015), schizophrenia (Perreault et al., 2010), and addiction (Perreault et al., 2016; Hasbi et al., 2018). However, despite several lines of evidence supporting the existence and function of D1-D2 heterodimers, evidence has also been presented against their existence in mouse brain, at least (Frederick et al., 2015).

#### Signal Transduction by Gβγ Subunits

Gβγ heterodimers interact with, and regulate, many GPCR effectors including multiple AC isoforms, ion channels and G protein-coupled receptor kinases (GRKs) (reviewed in Khan et al., 2013). Although much is known about what is *possible* for Gβγ-dependent signaling generally, little is known about which specific Gβγ-dependent events unfold following D1R activation in distinct neuronal populations. What is known is that in striatal neurons, the Gα_olf_ coupled D1R forms a specific complex with Gβ_2_γ_7_ and the formation of this heterotrimer is critical for the stability, trafficking and signaling of Gα_olf_ (Schwindinger et al., 2003, 2010; Hervé, 2011; Xie et al., 2015). Notably, the Gα_olf_β_2_γ_7_ heterotrimer forms a stable interaction with AC type 5 (AC5) under basal conditions, and this pre-assembled complex promotes AC5 protein stability and efficient activation of cAMP signaling by the D1R (Xie et al., 2015). Gβγ subunits are also known to interact with specific G protein-coupled receptor kinases (GRKs) (Lodowski et al., 2003a,b) leading to receptor phosphorylation, β-arrestin recruitment, and desensitization (Luttrell et al., 1999). As described in the following section, GRKs are critical regulators of D1R signaling, and although the role of Gβγ signaling to GRKs has not specifically been studied in the context of the D1R, data from work focused on other GPCRs suggests a potential role.

#### Receptor Phosphorylation, β-Arrestin Recruitment, and G Protein-Independent Signaling

In addition to stimulating G protein-dependent signaling, D1R activation can also promote phosphorylation of the receptor itself, leading to desensitization and internalization. The D1R can be phosphorylated on the carboxy-terminal tails or intracellular loops by protein kinases including PKA (Jiang and Sibley, 1999) and GRKs (Tiberi et al., 1996; Lamey et al., 2002; Kim et al., 2004; Sedaghat and Tiberi, 2011). Receptor phosphorylation creates or reveals a binding site for recruitment of β-arrestins (β-arrestin1, β-arrestin2), which desensitize the receptor and can initiate receptor internalization through clathrin-dependent endocytosis (Peterson and Luttrell, 2017). Consistent with this model of GRK function, genetic ablation of GRK2 in D1R-expressing striatal neurons enhances the locomotor-stimulating effects of cocaine and leads to increased phosphorylation of DARPP-32, a downstream target of PKA (Daigle et al., 2014). Similarly, in rodent models of Parkinson's Disease, GRK6 overexpression attenuates D1R sensitization, promotes D1R internalization and reduces the incidence of L-DOPA-induced dyskinesia (LID), an adverse effect associated with excessive activation of D1R-dependent signaling (Ahmed et al., 2010).

Phosphorylation of a GPCR promotes recruitment of β-arrestins, desensitization, and subsequent internalization of the GPCR or GPCR-arrestin complex. In the case of the D1R, desensitization is mediated preferentially by β-arrestin2 (Oakley et al., 2000) and internalization is transient, characterized by initial clathrin-dependent endocytosis followed by recycling to the cell surface (Dumartin et al., 1998; Vickery and von Zastrow, 1999; Martin-Negrier et al., 2006). β-arrestin recruitment can also initiate secondary, G protein-independent (or post-G protein) signaling pathways through scaffolding of various effector molecules. Although this second wave of signaling has been well-described for many GPCRs (reviewed in Jean-Charles et al., 2017), it has only recently begun to be characterized for the D1R. For example, in HEK 293 cells heterologously expressing the D1R, β-arrestin recruitment is promoted by specific GRK-dependent phosphorylation events, and β-arrestin recruitment subsequently contributes to the activation of extracellular signal-regulated kinases 1/2 (ERK1/2) synergistically with G protein-dependent mechanisms, and can activate Src kinase (independently of G proteins) (Kaya et al., 2020). To our knowledge, it remains unknown whether this β-arrestin-dependent signaling is recapitulated in neurons that endogenously express the D1R. However, there is some indirect evidence to support the existence of β-arrestin-dependent signaling by the D1R *in vivo*. For example, in one study acute morphine administration induced an interaction between β-arrestin2 and ERK1/2 in the nucleus accumbens, and this interaction was abolished in D1R-KO mice (Urs et al., 2011). One interpretation of this finding is that D1R signaling promotes ERK1/2 activation through a β-arrestin dependent mechanism, however this is far from conclusive and several alternative mechanisms could explain this observation. As discussed below, the activation of ERK1/2 by D1R signaling can occur through multiple mechanisms, including Gα_s/olf_-dependent signaling, so the relative contribution of β-arrestin remains unclear.

In the context of Parkinson's Disease, striatal β-arrestin2 was found to promote the therapeutic effects of L-DOPA treatment while reducing adverse effects such as dyskinesia (Urs et al., 2015; Zhang et al., 2019). Specifically, overexpression of β-arrestin2 in the striatum attenuated the development of LID whereas β-arrestin2 knockdown not only worsened LID (in both rodent and non-human primate models of PD) (Urs et al., 2015; Zhang et al., 2019) but also diminished the positive effects of L-DOPA on motor function (Urs et al., 2015). Increased D1R signaling is critically linked to the development of dyskinesia (Aubert et al., 2005; Pavón et al., 2006; Santini et al., 2007; Darmopil et al., 2009; Lebel et al., 2010; Alcacer et al., 2012), so it might be expected that facilitating D1R desensitization through overexpression of β-arrestin2 would attenuate LID. However, the observation that β-arrestin also contributed to the *therapeutic* effects of L-DOPA could be considered indirect evidence for the existence of therapeutically relevant β-arrestin-dependent signaling beyond its role in desensitization *per se*.

#### D1R Modulation Through Formation of Receptor Heteromers

Aside from the proposed D1-D2 heterodimer described above, the D1R can interact with a number of other cell-surface receptors, and such interactions have been reported to modify D1R signal transduction (reviewed in Perreault et al., 2014). Reported dimerization partners include the D2 (see above), D3 (Marcellino et al., 2008), H3 histaminergic (Ferrada et al., 2009; Moreno et al., 2011), mGluR5 (Sebastianutto et al., 2020), σ1 (Navarro et al., 2010), NMDA (Lee et al., 2002), and A1 adenosine (Ginés et al., 2000) receptors. While it is beyond the scope of this review to describe the specific signaling impacts for all these heterooligomers, many of these complexes have now been shown to exist *in vivo*, and context-dependent regulation of these complexes may help explain alterations in D1R signaling observed in different diseases. For example, the expression of the D3R in D1R-expressing striatal neurons is increased following dopamine depletion in animal models of Parkinson's Disease, and co-activation of the D3 receptor has been shown to modulate D1R signaling relevant to the development of LID (Solís et al., 2017; Lanza et al., 2018). Similarly, striatal dopamine depletion has been shown to increase the formation of D1R-mGluR5 heterodimers, leading to maladaptive signaling that promotes dyskinesia (Sebastianutto et al., 2020). While we are only beginning to understand the function of these various complexes, pharmacological targeting of specific receptor heterooligomers promises to be an intriguing area for future drug development (Ferré et al., 2021).

### Cell Type-Specific Aspects of D1R Signaling

#### cAMP/PKA Signaling in Cortical vs. Striatal Neurons

**Figure 3:**
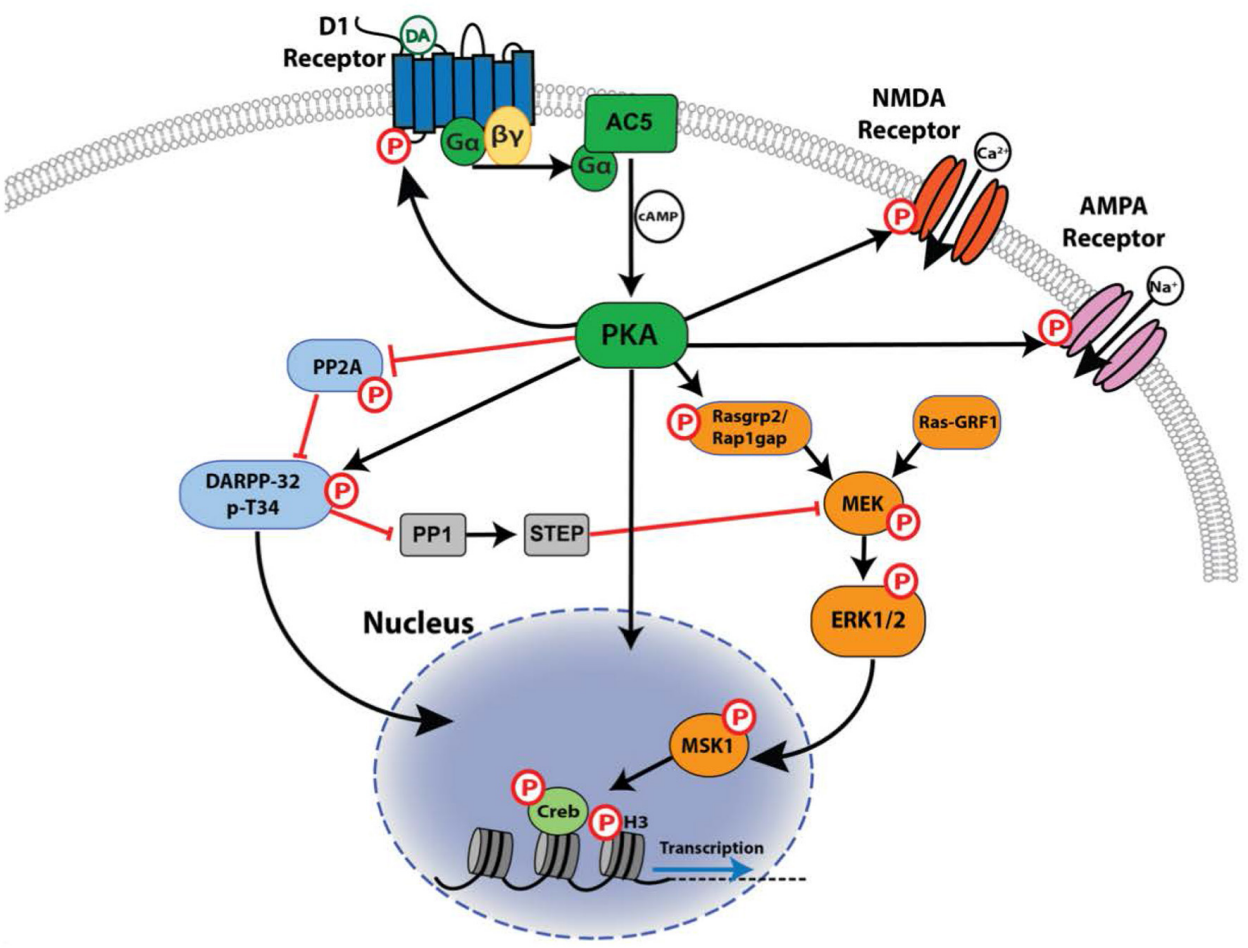
Signaling downstream of the D1R. Shown is the network of core signaling pathway interactions downstream of D1R activation in striatal neurons, with a focus on phosphorylation-dependent (P) actions mediated by PKA. Activation is indicated by a black arrowed line, inhibition by a red line.

D1R signaling through Gα_s/olf_ induces the activity of adenylyl cyclase, leading to the production of cAMP and subsequent activation of PKA. Once activated, PKA can regulate neuronal functions through direct phosphorylation of substrates in the cytosol, nucleus, and synaptic membranes, and can also initiate further downstream signaling through activation or inhibition of secondary effectors such as DARPP-32 (dopamine- and cAMP-regulated phosphoprotein of 32 kilodaltons), ERK1/2, and PP2A (protein phosphatase 2A) (Figure 3). Little is currently known about the contributions of non-PKA targets of cAMP in D1R-dependent signaling, despite the fact that proteins such as EPAC isoforms are highly enriched in the striatum, where D1R signaling plays an important role (Kawasaki et al., 1998).

The extent and kinetics of D1R-mediated cAMP/PKA activation are cell-type dependent. For example, while D1R activation stimulates cAMP production in both cortical and striatal neurons, higher cAMP concentrations and higher levels of PKA activity are generated in striatal neurons (Castro et al., 2013). Moreover, striatal neurons are sensitive to transient, sub-second pulses of extracellular dopamine to which these neurons respond with strong and long lasting cAMP/PKA signals, whereas cortical neurons require sustained receptor stimulation to produce comparable responses (Castro et al., 2013; Yapo et al., 2017). Activated PKA can translocate to the nucleus to regulate transcriptional events, and these regional or cell-type differences also extend to the nuclear compartment. For example, in cortical and thalamic neurons, nuclear PKA accumulation occurs slowly, and only when receptor stimulation is sustained (Gervasi et al., 2007; Hu et al., 2011). The temporal dynamics of nuclear PKA activation in these neurons is consistent with passive diffusion of the activated PKA catalytic subunit into the nuclear compartment. By contrast, in striatal neurons D1R stimulation produces a rapid, robust and sustained nuclear PKA signal that can be elicited even by a transient pulse of released dopamine (Yapo et al., 2018; Jones-Tabah et al., 2021). The rate of nuclear PKA activation in striatal neurons suggests an additional mechanism at play beyond passive subunit diffusion.

These differences between cortical and striatal neurons do not appear to be mediated by differences in the expression of the receptor itself, or by the relative activities of Gα_s_ vs. Gα_olf_ (Castro et al., 2013). Instead, striatal neurons appear to possess a unique complement of downstream signaling effectors and regulators that allow them to respond rapidly to transient dopamine signals. Higher levels of phosphodiesterase (PDE) activity in cortical neurons (in particular PDE4) help constrain and localize cAMP production, such that only a sustained activation of the receptor can produce a pronounced cAMP accumulation (Castro et al., 2013). Another difference is the high expression levels of DARPP-32 in the striatum (Ivar Walaas et al., 1983). DARPP-32 plays an important role in amplifying PKA activity by inhibiting protein phosphatase 1 (PP1), and thereby generating a positive-feedback loop that sustains PKA activity and prevents de-phosphorylation of PKA targets (Castro et al., 2013).

#### D1R Signaling Downstream of PKA: DARPP-32

As introduced in the preceding section, DARPP-32 is a target of PKA phosphorylation that plays a major role in regulating D1R signaling in the striatum (Ivar Walaas et al., 1983). While its expression is not restricted to the striatum, DARPP-32 is considerably more abundant in striatal medium-spiny neurons (MSNs) than in any other brain region or neuronal subtype, and is often used as a molecular marker for identification of these cells (Ivar Walaas et al., 1983). MSNs are broadly divided into two pathways, based on both anatomical and molecular features with the striatonigral “direct pathway” (dMSNs) expressing the D1R and the striatopallidal “indirect pathway” (iMSNs) expressing the D2R (Thibault et al., 2013). In striatal MSNs, DARPP-32 acts as a signaling “hub” and depending on its phosphorylation state can subserve many functions including acting as an inhibitor of protein phosphatase 1 (PP1) or as an inhibitor of PKA. The phosphorylation state of DARPP-32 *in vivo* is modified in a cell-type (dMSN vs. iMSN) specific manner by administration of D1/D5R agonists, psychostimulants, and antipsychotic drugs (Bateup et al., 2008). Although there are few pharmacological tools to manipulate DARPP-32 function directly, much has been learned through genetic manipulation. For example, targeted deletion of DARPP-32 from D1R-expressing MSNs results in impaired synaptic plasticity, reduced basal and cocaine-induced locomotion, and significant attenuation of L-DOPA induced dyskinesia in a Parkinsonian model (Bateup et al., 2010), underscoring its importance as a regulator of D1R signaling.

PKA phosphorylation of the Thr34 residue on DARPP-32 converts it into an inhibitor of protein phosphatase 1 (PP1) (Hemmings et al., 1984). PP1 dephosphorylates many PKA substrates, and thus the activation of DARPP-32 by Thr34 phosphorylation acts to amplify PKA activity, enhancing and prolonging PKA-dependent phosphorylation events. In the absence of dopamine signaling, DARPP-32 is phosphorylated on Thr75 by cyclin-dependent kinase 5 (CDK5), and this modification turns DARPP-32 into a competitive inhibitor of PKA (Bibb et al., 1999). PKA itself can relieve this inhibitory constraint by phosphorylating and thus activating protein phosphatase 2A (PP2A), which can then dephosphorylate the Thr75 residue of DARPP-32 (Nishi et al., 2000; Ahn et al., 2007). The combination of DARPP-32 phosphorylation at Thr34 and dephosphorylation at Thr75 generates a positive feedback loop that keeps basal PKA signaling suppressed, but potentiates D1R-mediated PKA signals, helping to explain the uniquely sensitive responses of striatal MSNs discussed above.

DARPP-32 is also subject to additional regulation via phosphorylation of Ser137 and Ser97/102 residues, mediated by casein kinases 1 and 2 (CK1, CK2), respectively (Girault et al., 1989; Desdouits et al., 1995). Ser137 phosphorylation prevents dephosphorylation of Thr34 by calcineurin (PP2B), while Ser97/102 phosphorylation enhances phosphorylation of Thr34 by PKA, and promotes nuclear translocation of DARPP-32 where it can inhibit nuclear PP1, preventing dephosphorylation of nuclear PKA substrates (Stipanovich et al., 2008). DARPP-32 also integrates input from non-dopaminergic signaling pathways. For example, glutamatergic signaling through AMPA and NMDA receptors has been shown to promote de-phosphorylation of Ser97, and promote shuttling of DARPP-32 from the nucleus back into the cytosol (Nishi et al., 2017).

#### D1R Signaling Downstream of PKA: Glutamate Receptor Subunits

While glutamatergic input provides the main excitatory stimulus that drives striatal neuron firing, dopamine signaling via D1R can act as a powerful modulator. Indeed, glutamate receptor subunits are among the best-characterized targets of PKA, providing a mechanism by which D1R signaling can regulate synaptic strength at glutamatergic synapses. In striatal neurons, PKA has been found to phosphorylate the GluR1 subunit of the AMPA receptor on its Ser845 residue (Snyder et al., 2000). This modification is associated with increased AMPA currents (Price et al., 1999) and increased trafficking of GluR1-containing AMPA receptors to the cell surface (Swayze et al., 2004). GluR1 phosphorylation is enhanced by PP1 inhibition, and the activation of DARPP-32 plays an important role in facilitating this modification. In addition to PKA, other protein kinases such as PKC and ERK1/2 can also mediate AMPA Ser845 phosphorylation in striatal neurons (Oh et al., 2013), and it is likely that crosstalk between signaling pathways mediated by dopamine and other neuromodulators regulate the overall phosphorylation state of GluR1. In the ventral striatum (nucleus accumbens), psychostimulants, D1/D5R agonists and natural rewards all induce pGluR1-Ser845, and this effect is associated with reward enhancement and facilitation of reward-related learning (Snyder et al., 2000; Carr et al., 2010). Striatal pGluR1 induced by psychostimulants is also abolished in D1R-KO mice, arguing that this pathway is mediated specifically by the D1R (Valjent et al., 2005).

In addition to modulating AMPA receptor function, D1R activation can also facilitate cortico-striatal plasticity via acute and long-term potentiation of NMDA receptor currents. NMDA receptor subunits are regulated by phosphorylation at multiple sites and by multiple protein kinases, including PKA (reviewed in Chen and Roche, 2007). Activation of the D1R leads to PKA-dependent phosphorylation of NR1 NMDA receptor subunits (Snyder et al., 1998) and phosphorylation-dependent trafficking of NR1 and NR2B subunits to the dendritic membrane in cultured striatal neurons (Hallett et al., 2006). This D1R-induced membrane trafficking enhances NMDA currents through a mechanism that involves both NR1 and NR2B subunits (Jocoy et al., 2011).

#### A Network Perspective of D1R Signaling Downstream of PKA

Many PKA targets exist beyond these well-described, canonical PKA substrates, and it is likely we have only scratched the surface of how PKA activity alters cellular function at the broader level of intracellular signaling networks. For example, a recent phospho-proteomic screen conducted in striatal tissue identified >200 proteins whose phosphorylation was increased following treatment with the adenylyl cyclase activator forskolin, and >100 proteins were phosphorylated following treatment with the D1/D5R agonist SKF 81297 (Nagai et al., 2016). Among the “hits” identified in this screen, PKA was found to phosphorylate RasGRP2 and Rap1GAP, which function to regulate the activity of the small GTPase Rap1 by acting as a guanine nucleotide exchange factor and GTPase-activating protein, respectively. PKA phosphorylation stimulates RasGRP2 while inhibiting Rap1GAP, the net effect being an increase in Rap1 activity. Activation of Rap1 in turn increases the excitability of striatal MSNs, and is an upstream activator of the mitogen-activated protein kinase kinase MEK, providing a previously unknown mechanism for PKA-dependent activation of ERK1/2 signaling (Nagai et al., 2016). As will be discussed in the following section, ERK1/2 is an important downstream effector of D1R signaling. The latter study illustrates the complexity of D1R- and PKA-dependent signaling networks and highlights how functionally important aspects of these pathways are still being uncovered.

#### D1R-Dependent Activation of MAPK; the Many Routes to ERK1/2 Activation

The extracellular-regulated kinases ERK1 and ERK2 are closely related members of the mitogen-activated protein kinase (MAPK) family and are important effectors of D1R signaling. The importance of ERK1/2 in striatal dopamine signaling was initially suggested by the observation that many drugs of abuse activate ERK1/2 in the rodent striatum (Berhow et al., 1996), a phenomenon found to be largely absent in D1R-KO mice (Valjent et al., 2000). Blocking ERK1/2 activation by inhibition of the upstream kinase MEK attenuated not only the early gene expression response to cocaine, but also acute cocaine-induced hyperlocomotion and conditioned place preference (Valjent et al., 2000). These findings implicated the ERK pathway, via the D1R, in mediating both the acute effects of cocaine, and long-term behavioral adaptations associated with repeated drug exposure. Since these initial studies, striatal ERK1/2 activation has been revealed as a common molecular mechanism underlying striatal adaptations induced by drugs of abuse (reviewed in Cahill et al., 2014; Sun et al., 2016), in LID (Pavón et al., 2006; Santini et al., 2007; Murer and Moratalla, 2011; Spigolon and Fisone, 2018) and in striatum-dependent learning and memory (reviewed in Shiflett and Balleine, 2011).

ERK1/2 are proximally activated by phosphorylation mediated principally by MEK, but the D1R-associated signaling cascades that ultimately lead to ERK1/2 activation are numerous. Although D1R activation alone can stimulate ERK1/2 both in cultured striatal neurons (Jones-Tabah et al., 2021) and certain striatal areas *in vivo* (Gerfen et al., 2002), maximal ERK1/2 activation in the rodent striatum has been reported to require concurrent activation of both D1 and NMDA receptors (Valjent et al., 2005). Mechanistically, activation of ERK1/2 requires phosphorylation by its upstream protein kinase MEK (Mao et al., 2004) which is in turn regulated by Ras-guanine nucleotide-releasing factor 1 (Ras-GRF1), a neuron-specific Ras activator required for subsequent activation of ERK1/2 by either D1R or NMDA receptors (Fasano et al., 2009, 2010; Cerovic et al., 2015). NMDA receptor signaling activates MEK through Ras-GRF1, but when this occurs in the absence of concurrent D1R stimulation, MEK activity remains tempered by the activity of phosphatases PP1 and STEP (striatal-enriched tyrosine phosphatase) (Saxena et al., 1999; Paul et al., 2000; Valjent et al., 2005). D1R signaling on the other hand leads to DARPP-32 phosphorylation on Thr34, as discussed above, which leads to inhibition of PP1 and STEP, thereby disinhibiting and potentiating MEK/ERK1/2 activation (Paul et al., 2003; Valjent et al., 2005). Thus, simultaneous activation of MEK by NMDA receptors, together with PP1/STEP inhibition mediated by D1R signaling, leads to the amplified ERK1/2 activity induced by many abused drugs (Halpain et al., 1990; Nishi et al., 2000; Valjent et al., 2005). Consistent with this role of ERK1/2 as a “coincidence detector,” stimulation of several of the D1R heteromers described in section 2.1.5 appear to activate ERK1/2 above what can be accomplished by the component monomeric receptors alone (Ferrada et al., 2009; Moreno et al., 2011; Lanza et al., 2018; Sebastianutto et al., 2020), with the specific mechanisms varying between the different heterooligomeric complexes.

Striatal activation of D1R-dependent ERK1/2 signaling is also causally implicated in development of LID, although here the mechanism of ERK1/2 activation differs from that observed with drugs of abuse (reviewed in Spigolon and Fisone, 2018). In animal models of Parkinson's Disease where dopaminergic neurons are largely destroyed and striatal dopamine depleted, D1R signaling becomes sensitized; this sensitization when combined with chronic L-DOPA treatment results in dyskinesias, i.e., LID (Aubert et al., 2005). In such animal models, administration of either L-DOPA or D1/D5R agonists potently activates both cAMP/PKA-dependent signaling and subsequently ERK1/2 activation, to significantly greater extents than in the absence of a dopamine lesion (Gerfen et al., 2002; Pavón et al., 2006; Santini et al., 2007; Westin et al., 2007; Darmopil et al., 2009; Jones-Tabah et al., 2020) and this phenomenon is abolished in D1R- but not D2R-KO mice (Darmopil et al., 2009). The hypothesis that increased coupling of D1R to the ERK1/2 pathway is a causal factor leading to LID is supported by the observation that inhibition of upstream regulators like MEK (Santini et al., 2007) or Ras-GRF (Fasano et al., 2010) attenuate dyskinesia. In contrast, genetic enhancement of Ras activity failed to potentiate either LID or ERK1/2 activation in a mouse model of LID, suggesting that in this model, ERK1/2 activity may approach a physiological maximum following chronic L-DOPA (Ruiz-DeDiego et al., 2018). The exact mechanism of this increased coupling of D1R to ERK1/2 remains under investigation but appears to depend in part on canonical D1R signaling through Gα_olf_ and PKA, as follows. Heterozygous knockout of Gα_olf_ in the striatum attenuates PKA-dependent phosphorylation of GluA1 and DARPP-32, but not ERK1/2 activation in LID (Alcacer et al., 2012). In contrast, complete Gα_olf_ knockout or PKA inhibition does prevent ERK1/2 activation (Lebel et al., 2010; Alcacer et al., 2012). In other words, ERK1/2 activation in LID is dependent on, but not linearly related to upstream Gα_olf_/PKA activity, suggesting that in the dopamine depleted striatum, ERK1/2 may become maximally activated with even low levels of Gα_olf_/PKA signaling, or that multiple convergent mechanisms lead to ERK1/2 activation in LID. Indeed, additional LID-specific mechanisms of D1R-dependent ERK1/2 activation have been described (Sebastianutto et al., 2020). These include an interaction of the D1R with the Src homology 2 domain-containing phosphatase 2 (SHP2), leading to SHP2-dependent disinhibition of Src kinase, an upstream regulator of ERK1/2 (Fiorentini et al., 2011). The D1R/SHP2 pathway is persistently activated in LID (Fiorentini et al., 2013), and knockdown of SHP2 significantly attenuates both L-DOPA-induced ERK1/2 activation and behavioral manifestations of dyskinesia in rodent models (Fiorentini et al., 2016). In the striatum of dopamine depleted rats, inhibition of Src was also found to attenuate D1R-mediated ERK1/2 activation (Fieblinger et al., 2014), reinforcing the possibility that Src may play an important role as an upstream regulator of ERK1/2, leading to the development of LID.

Similar to PKA, ERK1/2 kinases have many cellular substrates in synaptic membranes, the cytosol and nucleus, but the relative importance of individual targets to overall alterations in neuronal function remain incompletely understood. Here we will highlight a few of the known targets and the diverse cellular processes they regulate. ERK1/2 activity regulates long term potentiation (LTP) at cortico-striatal synapses, and cocaine, for example, drives NMDA-dependent LTP in a D1R- and ERK1/2-dependent manner (Pascoli et al., 2012). This cocaine-mediated LTP is associated with increased AMPA receptor expression at the cell surface, an effect that appears to be mediated by ERK1/2-dependent phospho-inhibition of phosphodiesterase 4 (PDE4) at the synapse; PDE4 inhibition in turn facilitates local PKA activation, leading to increased AMPA receptor insertion into the membrane facilitated by GluR1 phosphorylation (Song et al., 2013).

Another important ERK1/2 target is the *mechanistic target of rapamycin* (mTOR) complex 1 (mTORC1), a regulator of mRNA translation whose activation by ERK1/2 plays a role in the regulation of LTP and memory encoding (Kelleher et al., 2004), and in cocaine-induced locomotor sensitization (Wu et al., 2011). In rodent models of LID, mTORC1 is activated in D1R-expressing neurons through a mechanism involving both DARPP-32 and ERK1/2, and inhibition of mTORC1 or its upstream activators prevented the development of dyskinesia (Santini et al., 2009, 2012). ERK1/2 signaling also plays an important role in regulation of transcriptional responses through direct phosphorylation of nuclear targets and the activation of downstream nuclear protein kinases such as mitogen- and stress-activated protein kinase 1 (MSK1) and ribosomal S6 kinase 1 (RSK1) (Xing et al., 1996), which will be discussed in the following section.

### Nuclear Signaling Downstream of the D1R

As with many GPCRs, D1R signaling initiated at the cell surface can propagate to other cellular compartments, including the nucleus. Several D1R effectors including PKA, ERK1/2 and DARPP-32, can be translocated to the nucleus, where they mediate phosphorylation of nuclear targets and regulate gene expression (Girault et al., 1989; Nishi et al., 2000; Stipanovich et al., 2008). Signaling-dependent gene expression plays a role in regulating striatal plasticity, learning, and memory, as revealed through the use of transcriptional or protein synthesis inhibitors (Hernandez et al., 2002; Yin et al., 2006; Piechota et al., 2010; Jonkman and Everitt, 2011; Luo et al., 2011). In the context of dopaminergic pharmacology, abnormal D1R-dependent regulation of gene-expression contributes to the development of maladaptive drug responses such as LID. While the importance of this transcriptional output has been well-established, the specific signaling mechanisms that link D1R activation at the cell surface to transcriptional and epigenetic regulators in the nucleus are complex, and our understanding of these processes continues to evolve.

#### Nuclear PKA Signaling

As described previously, after activation of D1Rs in cortical and striatal neurons, PKA can translocate to (or become activated within) the nucleus, but to different extents and with different kinetics (Yapo et al., 2018; Jones-Tabah et al., 2021). Specifically, in cortical neurons, the activity of nuclear PKA increases slowly, and only upon sustained D1R stimulation. However, in striatal neurons nuclear PKA is activated rapidly, and requires only a transient D1R stimulus. Nuclear substrates for PKA-dependent signaling have been identified in various cell types, yet because PKA activates and co-signals alongside other protein kinases in the nucleus, there is limited knowledge about which targets are acted on directly by PKA. The most well-characterized nuclear target of PKA is the cAMP response element binding protein (CREB), a transcription factor which binds to DNA sequences known as cAMP response elements and plays a critical role in regulating many activity-regulated genes. In many cell types PKA has been shown to phosphorylate CREB on S133 but many other protein kinases can also regulate CREB activity (Lin et al., 1998; Delghandi et al., 2005; Naqvi et al., 2014), and in striatal neurons, there is currently a lack of evidence supporting direct regulation of CREB by PKA. Instead, it seems that in striatal neurons, the downstream protein kinase MSK1 (discussed below) plays an obligate role in facilitating CREB phosphorylation and may indeed be the major regulator (Brami-Cherrier et al., 2005).

Nuclear PKA signaling can also activate transcription through de-repression of target genes via activation of αCREM (cAMP response element modulator), which antagonizes transcriptional repression mediated by DREAM (downstream regulatory element antagonistic modulator) (Ledo et al., 2000). Activation of this PKA/αCREM/DREAM pathway appears to play a role in the D1R-dependent transcriptional activation that contributes to L-DOPA induced dyskinesia (Ruiz-DeDiego et al., 2015a). Besides transcriptional regulators such as CREB and CREM, PKA has also been shown to regulate nuclear function through targeting histone modifying enzymes including histone deacetylases (Sunagawa et al., 2010) and histone demethylases (Baba et al., 2011). However, the role of these targets downstream of D1R signaling has not specifically been explored.

#### Nuclear ERK1/2 Signaling

Upon activation, phosphorylated ERK1/2 can translocate to the nucleus, a response well-documented in striatal neurons (Sgambato et al., 1998). In cultured striatal neurons, both PKA and ERK1/2 are activated in the nucleus with similarly rapid kinetics following D1R activation (Jones-Tabah et al., 2021). The nuclear targets of ERK1/2 include transcription factors such as CREB and Elk-1 (Valjent et al., 2001), and nuclear protein kinases such as MSK1 and RSK (Xing et al., 1996). Preventing ERK1/2 activation via inhibition of MEK largely attenuates transcriptional activation induced by the D1R in striatal neurons (Savell et al., 2020). The regulation of transcription in striatal neurons by ERK1/2 has received considerable attention due to its relevance to drug abuse and LID. Specifically, ERK1/2 is a key regulator of immediate early genes (IEGs) such as Fos, FosB, Arc, and Egr1/zif268, whose induction contributes to striatal adaptations associated with these disorders (Andersson et al., 1999; Valjent et al., 2006; Darmopil et al., 2009; Carta et al., 2010; Chen et al., 2017).

#### Nuclear MSK1 Signaling

One of the best-characterized nuclear targets of ERK1/2 is the mitogen- and stress-activated protein kinase 1 (MSK1), a nuclear protein kinase which plays an important role in activating CREB and regulating post-translational chromatin modifications (Deak et al., 1998). Nuclear MSK1 signaling in D1R-expressing striatal neurons contributes to the rewarding effects of cocaine (Brami-Cherrier et al., 2005) as well as the development of LID (Feyder et al., 2016). In the striatum, MSK1 contributes to phosphorylation of CREB (Brami-Cherrier et al., 2005) and mediates phosphorylation of histone H3 on Ser10 (abbreviated as H3S10p), a post-translational chromatin modification associated with transcriptional activation (Clayton et al., 2000). MSK1 knockout has functional consequences; although it did not affect acute locomotor responses to cocaine, it did prevent induction of several IEGs, and markedly reduced locomotor sensitization to repeated cocaine administration (Brami-Cherrier et al., 2005). Similarly, MSK1 knockout did not alter the acute anti-Parkinsonian effects of L-DOPA in 6-OHDA lesioned mice, but attenuated induction of FosB and development of LID after sustained L-DOPA treatment (Feyder et al., 2016). These studies suggest that nuclear signaling by MSK1, although not required for the acute effects D1R stimulation, is required for long-term adaptations which underpin the development of pathological states such as addiction or LID.

In striatal neurons, MSK1 has also been shown to mediate phosphorylation of H3 on Ser28 (H3S28p), specifically in the context of trimethylated H3K27me3-containing nucleosomes. H3K27me3 is an inhibitory chromatin modification which is mediated in part through the recruitment of polycomb group proteins. However, H3S28p is able to displace the polycomb proteins, leading to de-repression of polycomb target genes (Gehani et al., 2010; Lau and Cheung, 2011). *In vivo*, MSK1-dependent phosphorylation of H3S28 has been shown to occur in D1R-expressing striatal neurons either in response to amphetamine (Bonito-Oliva et al., 2016) or after L-DOPA treatment in animals that develop LID (Södersten et al., 2014). In this latter example, aberrant transcriptional activation of polycomb target genes was also observed and proposed to contribute to maladaptive plasticity associated with LID.

#### Transcriptional and Epigenetic Regulation by D1R

The nuclear targets of the protein kinases described in the preceding sections include transcription factors, histones, chromatin remodelers and DNA modifying enzymes, which generally function to regulate transcription and co-transcriptional processes. Many transcription factors have been identified to play a role in mediating gene expression downstream of the D1R, and these include SRF (Parkitna et al., 2010), NPAS2 (Parekh et al., 2019), NPAS4, (Funahashi et al., 2019), MEF2A (Pulipparacharuvil et al., 2008), NFκB (Russo et al., 2009), and zif268/EGR1 (Carta et al., 2010). However, the most extensively characterized nuclear target associated with D1R signaling is the transcription factor CREB.

CREB is activated by phosphorylation on Ser133, which facilitates its interaction with co-activators like CREB-binding protein (CBP, also called p300) (Goodman and Smolik, 2000) or cAMP-regulated transcriptional coactivators (CRTCs) (Altarejos and Montminy, 2011). As described previously, CREB phosphorylation on Ser133 can be mediated by many protein kinases including PKA, ERK1/2, and MSK1. In striatal neurons, although PKA activation can lead to CREB phosphorylation, MSK1 likely mediates the bulk of CREB phosphorylation in response to psychostimulant (Brami-Cherrier et al., 2005) or L-DOPA treatment (Feyder et al., 2016), and PKC- and ERK1/2-dependent mechanisms have also been described (Zanassi et al., 2001).

Genes induced by stimulation of the D1R are highly enriched in DNA binding motifs for CREB and D1R-dependent transcriptional activation is largely attenuated by CREB inhibition (Savell et al., 2020). CREB function has been shown to regulate excitability of striatal MSNs (Dong et al., 2006), behavioral responses to cocaine (Carlezon et al., 1998), and striatal-dependent memory (Pittenger et al., 2006). The genomic targets of CREB include prototypical immediate-early genes such as *Fos* as well as many neuropeptides and signaling proteins important in neuronal function and plasticity. CREB activation plays an important role in mediating LTP and genetic depletion of either CREB or the co-activator CBP impair striatal plasticity, learning, and cocaine associated gene expression (Pittenger et al., 2006; Malvaez et al., 2011). In fact, analysis of genome-wide transcriptional changes induced by cocaine self-administration (Walker et al., 2018) or during L-DOPA treatment leading to LID (Heiman et al., 2014) have shown that induction of CREB-regulated genes plays a predominant role in molecular adaptations apparent in both these conditions.

In addition to transcription factors, D1R signaling also regulates epigenetic modifiers which can mediate alterations in chromatin structure, DNA methylation, or histone post-translational modifications. As mentioned above, D1R signaling is able to induce phosphorylation of histone H3 through ERK/MSK1-dependent signaling. H3 phosphorylation at the promoter of genes such as *Fosb* induces transcription and is a required step in the development of LID (Feyder et al., 2016). Similarly, acute psychostimulant exposure induces deposition of another activating histone modification, acetylation of histone H4, mediated in this case by CBP. In this case, H4 acetylation by CBP at the *Fosb* promoter induces expression and accumulation of FosB protein, thought to be a critical step in development of sensitized drug responses and behaviors associated with addiction (Levine et al., 2005). The expression of FosB can also cause further alteration of the chromatin landscape. For example, chronic cocaine-induced FosB accumulation mediates repression of the histone lysine dimethyltransferase G9a, leading to loss of the repressive histone mark H3K9 dimethylation (Maze et al., 2010). This serves as an illustration of how signaling events can cascade through multiple levels of genomic regulation to produce long-lasting changes in gene expression. In fact, exposure to cocaine causes widespread alterations in histone acetylation and methylation at thousands of loci across the genome (Renthal et al., 2009) and the role of specific alterations in modulating behavior are only starting to be understood.

DNA methylation levels are known to be altered both in response to drugs of abuse (Massart et al., 2015), and in LID (Figge et al., 2016). D1R stimulation has specifically been shown to rapidly upregulate expression of the DNA demethylase Gadd45b both in primary cultures and *in vivo* (Savell et al., 2020; Zipperly et al., 2021). Knockdown of Gadd45b impairs D1R-dependent changes in DNA methylation, which appear to be required for receptor-driven transcriptional activation, as well as cocaine reward (Zipperly et al., 2021). Gadd45b is similarly upregulated in LID, but interestingly, knockout of Gadd45b worsens the development of dyskinesia and increases the expression of other D1R-regulated genes such as *Fos* and *Fosb* (Park et al., 2016), suggesting that the demethylase may play distinct roles in different striatal subregions, or depending on specific disease context.

### Perspectives

The signaling pathways regulated by the D1R have been extensively investigated, and a full accounting of the cellular functions affected by these pathways would exceed the scope of any single review. We have here presented a summary of critical features of D1R-directed signaling, but it is important to recognize that many of these features describe D1R signaling as it occurs in select contexts. For example, a majority of D1R-signaling studies have been performed with a focus on the striatum, and the D1R-expressing medium-spiny neurons found there. We are only beginning to understand how these processes differ between neuronal subtypes. It is also clear that while much is known about the activation of relatively proximal signaling effectors like the kinases PKA and ERK1/2, much less is known about how these effectors then alter cellular functions at a network level. An emerging challenge will be to match our deepening understanding of signaling with recent advances in drug development that will be discussed in the following sections, and would allow us to specifically target therapeutically relevant aspects of the signaling network induced by D1R activation.

### D1R Pharmacology in Neuropsychiatric Disease: Focus on Parkinson's Disease and Cognitive Impairment

Although dysregulation of D1R signaling has been implicated in numerous neurodegenerative and neuropsychiatric conditions, the principle indications for which clinical use of D1R ligands has been studied are Parkinson's Disease (PD) and cognitive impairment. Below, we briefly summarize preclinical and clinical studies pertaining to the use of D1R ligands in the treatment of these conditions.

### D1/D5R Agonists as Therapeutics in Parkinson's Disease

#### Clinical and Pathophysiological Features of Parkinson's Disease

Parkinson's Disease is the second most common neurodegenerative disorder, affecting ~1–2% of the population aged 65 years or older (de Lau and Breteler, 2006; Hirsch et al., 2016). While the specific causes of PD are numerous, PD pathology is characterized by progressive, and ultimately substantial, degeneration of the nigrostriatal dopaminergic pathway (Poewe et al., 2017). This loss of dopaminergic neurons leads to depletion of striatal dopamine and causes the hallmark motor symptoms of PD: tremor at rest, rigidity, bradykinesia, and postural instability (Postuma et al., 2015). Other non-motor symptoms such as cognitive and neuropsychiatric dysfunction may also occur, particularly in later stages of the disease as degeneration spreads to additional brain structures (Schapira et al., 2017).

#### Pharmacotherapy for Parkinson's Disease

The mainstay of pharmacological management of PD motor symptoms is L-3,4-dihydroxyphenylalanine (L-DOPA), a dopamine precursor which bolsters endogenous synthesis of dopamine. Introduced in the late 1960s, L-DOPA remains both the most effective and most commonly used treatment of PD. Due to its conversion to dopamine, L-DOPA administration results in a stimulation of both D1-class and D2-class receptors. However, while L-DOPA is effective in alleviating motor symptoms in early stages of PD, there are major limitations to its long term use. These include reduced efficacy, due to progressive degeneration of the neurons that synthesize and release dopamine, and the development of involuntary movements generally referred to as L-DOPA-induced dyskinesia (LID) (Espay et al., 2018). Approximately 50–80% of PD patients develop dyskinesia after prolonged L-DOPA treatment (Thanvi et al., 2007). LID can represent a major source of disability and a predominant dose-limiting factor in the use of L-DOPA. In extreme cases of LID, patients cycle between ON responses complicated by severe dyskinesia and OFF responses with disabling Parkinsonism (Thanvi et al., 2007). Long-acting controlled release preparations of L-DOPA have increased the overall effectiveness of the drug by reducing the fluctuations in plasma levels, which have been associated with the development of LID (Huot et al., 2013).

While L-DOPA remains the gold standard treatment for motor symptoms in PD, first-line therapies also include agonists that directly target dopamine receptors, and these present several clinical advantages. While L-DOPA needs to be metabolized into dopamine and released from presynaptic terminals, dopamine agonists can directly activate post-synaptic receptors, bypassing the need for a presynaptic release. Dopamine agonists also have longer half-lives and longer duration of action than L-DOPA, leading to reduced motor fluctuations. However, dopamine agonists currently used in PD treatment target primarily the D2 receptor, and are generally not as efficacious as L-DOPA (Poewe et al., 2017).

In animal models of PD, agonists that target D1 receptors have been found to alleviate motor impairment (Temlett et al., 1988, 1989; Emre et al., 1992; Kebabian et al., 1992a; Goulet and Madras, 2000), and in non-human primates, D1R agonists have been shown to be particularly effective in treating advanced Parkinsonism (Goulet and Madras, 2000), which remains particularly difficult to treat with currently available therapies. Thus, as described above, in many cases the failure of preclinical development of D1R agonists has been attributed not to invalidity of this therapeutic strategy, but to the pharmacokinetic limitations and preponderance of adverse effects associated with the specific ligands (Temlett et al., 1988, 1989; Emre et al., 1992; Kebabian et al., 1992a; Blanchet et al., 1998; Rascol et al., 1999; Salmi et al., 2004). The recent development of non-catecholamine D1/D5R agonists has renewed interest in their therapeutic development for management of PD. In the following sections, we will outline preclinical and clinical evidence supporting the use of D1/D5R agonists in PD treatment, and important considerations pertaining to the risk of dyskinesia presented by the long-term use of D1/D5R agonists.

#### D1/D5R Agonists in PD: Benzazepines

The 1-phenylbenzazepine scaffold was one of the earliest used for the synthesis of D1/D5R-selective ligands and produced a wide range of pharmacological agents (i.e., full agonists, partial agonists, antagonists; Neumeyer et al., 2003; Giri et al., 2020). The most widely-studied benzazepine D1/D5 ligand is the partial agonist SKF 38393. Despite having anti-Parkinsonian effects in 6-OHDA-lesioned rodents (Arnt and Hyttel, 1985), this drug failed to improve motor function in either MPTP-treated monkeys or PD patients, either when given alone or in combination with L-DOPA (Braun et al., 1987; Bedard and Boucher, 1989). CY-208243 was another early benzazepine D1/D5 agonist that proved insufficiently effective in patients (Tsui et al., 1989) despite showing promising anti-Parkinsonian effects in marmosets (Temlett et al., 1989). Another benzazepine, SKF 81297 is a full D1/D5R agonist found to stimulate motor function, either alone or in combination with a D2R agonist in MPTP-lesioned rhesus monkeys (Vermeulen et al., 1993, 1994). A highly related compound, fenoldopam, is FDA-approved for emergency treatment of hypertensive crises, but does not cross the blood brain barrier (Hahn et al., 1982). Although many benzazepine derivatives were developed and some showed promising preclinical potential for PD, poor oral bioavailability, propensity to cause seizures, and poor penetration of the blood brain barrier limited the therapeutic development of these agonists.

Interestingly, several benzazepine agonists have been reported to act as biased agonists. For example, a few benzazepines, including SKF 38393 and SKF 83959 appear to act as G protein-biased ligands that do not stimulate β-arrestin recruitment in cells heterologously expressing the D1R (Conroy et al., 2015). Other studies have found either that SKF 83959 has a distinctive ability to stimulate phospholipase C downstream of the D1R (Jin et al., 2003) or to act as a specific agonist of D1-D2 heterodimers (Rashid et al., 2007). In rodent models of PD, SKF 83959 was found to have promising anti-Parkinsonian effects, stimulating motor function and causing minimal dyskinesia. Moreover, co-treatment with SKF 83959 alongside L-DOPA attenuated the development of LID (Zhang et al., 2007). Unfortunately, although SKF 83959 has attracted considerable attention for its unique pharmacology, unraveling the origin of its behavioral effects is complicated by the fact that this drug has appreciable affinity for additional targets including σ receptors, α-adrenergic receptors, and serotonin receptors (Chun et al., 2013; Guo et al., 2013), and no follow-up studies have yet investigated the underlying mechanism of its promising anti-Parkinsonian profile.

#### D1/D5R Agonists in PD: Dihydrexidine

Dihydrexidine was reported to be the first high potency, D1/D5R full agonist that crossed the blood brain barrier (Lovenberg et al., 1989; Salmi et al., 2004), and exhibited promising anti-Parkinsonian effects in rodent and non-human primate models of PD (Bedard and Boucher, 1989; Watts et al., 1993). Although dihydrexidine has some D2R affinity, its anti-Parkinsonian effects were blocked by the D1/D5R antagonist SCH23390 but not the D2R antagonist remoxipride (Mottola et al., 1992) providing a critical link that D1R activation was a viable therapeutic strategy in PD. Dihydrexidine advanced to clinical trials, however its anti-Parkinsonian effects in patients were disappointing and the trial was hampered by poor bioavailability, and dose-limiting adverse effects including hypotension, and tachycardia (Blanchet et al., 1998). Interestingly, it was recently reported that although dihydrexidine acted as a full agonist at Gα_s_-coupled D1Rs, it was a partial agonist at Gα_olf_-coupled D1Rs which predominate in the striatum (Yano et al., 2018). Given that striatal D1Rs are the principal target for management of PD, this may have contributed to the failure of dihydrexidine.

Several analogs of dihydrexidine have now been developed in the hopes of improving the pharmacokinetic profile of the parent compound. Some such analogs demonstrated greater anti-Parkinsonian activity (Martin, 2011; McCorvy et al., 2012), however none have progressed through clinical trials. Pro-drugs were also proposed to enhance lipophilicity and penetration of the blood brain barrier (Sozio et al., 2012). ABT-431, the prodrug form of A-86929, is similar in structure to dihydrexidine (Rascol et al., 1999) and showed anti-Parkinsonian efficacy comparable to L-DOPA in PD patients, while producing reduced dyskinesia (Giardina and Williams, 2001; Rascol et al., 2001). Moreover, compared to dihydrexidine, ABT-431 was well-tolerated in PD patients with less hypotension reported and longer duration of action. Despite showing promise, ABT-431 was ultimately found to have poor oral bioavailability and needed to be given intravenously, severely limiting its potential use outside the hospital setting.

#### D1/D5R Agonists in PD: Benzazepine Derivatives

A-68930 and A-77636 are full D1/D5R agonists derived from the benzazepine scaffold and developed to overcome the poor oral bioavailability of dihydrexidine. Both compounds showed anti-Parkinsonian effects in 6-OHDA lesioned rats and MPTP lesioned non-human primates (Kebabian et al., 1992a,b). Moreover, A-68930 and A-77636 were less liable to produce dyskinesia in L-DOPA-primed MPTP-lesioned marmosets while maintaining anti-Parkinsonian efficacy (Pearce et al., 1999). Despite showing promise, both compounds produced rapid tolerance both in animals and patients (Kebabian et al., 1992a; Asin and Wirtshafter, 1993), and A-68930 also induced seizures (Kebabian et al., 1992b). Thus, although A-68930 and A-77636 showed greater bioavailability and improved anti-Parkinsonian activity compared to previous generations of D1R agonists, they were ultimately not advanced into further clinical trials.

#### D1/D5R Agonists in PD: Non-catechol Agonists

The recent development of non-catechol agonists with improved drug-like properties (Gray et al., 2018) has renewed clinical interest in the D1R as a therapeutic target for PD treatment. Indeed, at the time of writing, three agonists derived from this non-catechol scaffold have progressed into clinical development, where they have been found to be safe, well-tolerated and free from the peripheral side effects characteristic of other ligands (Gurrell et al., 2018; Papapetropoulos et al., 2018; Sohur et al., 2018). One such non-catechol ligand, PF-06649751, a G protein-biased partial agonist (later renamed CVL-751 and branded as Tavapadon), had earlier been found to have significant anti-Parkinsonian activity in non-human primates (Young et al., 2020). In a subsequent randomized placebo-controlled clinical trial conducted in patients with early stage PD, Tavapadon was further shown to be effective in relieving motor symptoms with once daily oral dosing (Riesenberg et al., 2020). Tavapadon has now progressed into Phase 3 clinical trials for treatment of PD motor symptoms. A related ligand, PF-06412562, has also been evaluated in a feasibility study and found to be well-tolerated in patients with advanced PD (Huang et al., 2020). Two other non-catechol partial agonists have been evaluated but to date have shown only limited efficacy (Gurrell et al., 2018; Papapetropoulos et al., 2018) and have not progressed to further clinical trials. In addition to having improved pharmacokinetic properties, these ligands do not stimulate the recruitment of β-arrestin, and appear not to desensitize the D1 receptor, suggesting they could avoid the rapid tolerance observed with typical D1/D5R full agonists.

#### D1R Signaling and the Development of LID

The development of non-catecholamine scaffolds has circumvented the pharmacokinetic obstacles that precluded the clinical use of D1R agonists. However, an additional obstacle to the use of D1R agonists in the treatment of PD is the established association between striatal dopamine depletion, D1R sensitization, and the subsequent development of LID upon treatment with L-DOPA or D1/D5R agonists. Although preclinical and clinical tests have generally supported the argument that D1/D5R agonism is an effective therapeutic strategy for alleviating motor impairment, D1R agonists still tend to produce similar levels of dyskinesia as L-DOPA (Temlett et al., 1988, 1989; Emre et al., 1992; Kebabian et al., 1992a; Blanchet et al., 1998; Goulet and Madras, 2000). Moreover, in MPTP-treated monkeys, D1/D5R antagonism concurrent with L-DOPA treatment prevents dyskinesia, but also impairs the therapeutic effects of L-DOPA (Grondin et al., 1999), suggesting that activation of the D1R is linked to both therapeutic and adverse outcomes. From animal models it has been established that following a period of striatal dopamine depletion, D1R signaling becomes sensitized, and that upon L-DOPA treatment, activity in the striatal direct pathway becomes progressively dysregulated (Ryan et al., 2018). In the DA-depleted striatum, D1R sensitization itself helps to maintain D1R signaling tone. However, upon treatment with L-DOPA or D1R agonists, hyperactivation of specific signaling pathways downstream of sensitized D1Rs sets off a chain reaction of striatal adaptations that ultimately lead irreversibly to dyskinesia. The evidence underlying these conclusions is summarized below.

D1R hypersensitization appears to be a significant factor underlying the development of LID in animal models (Aubert et al., 2005). Several factors can contribute to D1R hypersensitization in animal models of PD, including upregulation or altered trafficking of the D1R itself or its associated signaling partners such as Gα_olf_ (Penit-Soria et al., 1997; Corvol et al., 2004; Aubert et al., 2005; Alcacer et al., 2012; Morigaki et al., 2017) and AC5 (Rangel-Barajas et al., 2011). In PD patients, D1R sensitization also seems to occur, based on several observations from post-mortem human brain, including an increase in Gα_olf_ expression levels (Hurley et al., 2001; Corvol et al., 2004). In animal models of PD, this sensitization process leads to an intracellular “re-wiring” that increases the ability of the D1R to couple to the ERK1/2 pathway (Santini et al., 2007). The outcome of this sensitization is that D1R agonists gain the ability to activate cAMP/PKA and ERK1/2 signaling (Santini et al., 2007; Westin et al., 2007; Jones-Tabah et al., 2020). Stimulation of hypersensitized D1Rs in the dopamine-depleted striatum also results in robust phosphorylation of DARPP-32, and the activation of ERK1/2 and transcriptional signaling that induces the expression of immediate early genes such as ΔFosB (Hakansson et al., 2004; Pavón et al., 2006; Santini et al., 2007; Darmopil et al., 2009). Furthermore, abnormal PKA/DARPP-32 signaling increases the phosphorylation of GluR1 subunit of AMPA receptors, promoting the excitability of the striatal direct pathway (Snyder et al., 2000; Santini et al., 2007).

The causal role of this signaling in mediating the behavioral manifestation of LID has been established through a variety of pharmacological and genetic manipulations. In relation to specific downstream signaling events, targeting Gα_olf_-dependent signaling by knockout of AC5 or inhibition of PKA appear to attenuate dyskinesia, without affecting the therapeutic activity of L-DOPA (Lebel et al., 2010; Park et al., 2014). These findings suggest that cAMP-mediated signaling has a specific dyskinesiogenic effect. Reinforcing this possibility, chemogenetic stimulation of striatal direct-pathway neurons using DREADDs (*Designer Receptor Exclusively Activated by Designer Drugs*) in 6-OHDA lesioned mice promoted motor recovery with minimal dyskinesia when a Gq-DREADD (which activates neurons via calcium release) was used but caused severe dyskinesia when a Gs-DREADD (which activates cAMP signaling) was used (Alcacer et al., 2017). This finding further suggests that although activation of D1R-expressing striatal neurons promotes motor recovery, activation of cAMP signaling in this neuronal population produces dyskinesia.

Further downstream within the D1R signaling cascade, inhibition of ERK1/2 via targeting several of its upstream regulators (MEK, Ras-GRF, or RasGRP1) prevented the development of LID (Santini et al., 2007; Fasano et al., 2010; Eshraghi et al., 2020). However, it is noteworthy that MEK inhibition has also been found in at least one study to attenuate the *therapeutic* effect of L-DOPA (Urs et al., 2015). As introduced in section 2.3.2, once activated, ERK1/2 moves to the nucleus where it can activate MSK1 to regulate transcription through phosphorylation of CREB and histone H3 (Alcacer et al., 2014; Feyder et al., 2016). Like inhibition of PKA and ERK1/2, genetic ablation of MSK1 also attenuates LID and blocks the increased expression of ΔFosB (Feyder et al., 2016). The accumulation of ΔFosB itself also appears to be a causal factor in LID, since blocking the activity of ΔFosB can prevent LID development, or can even reverse established LID (Chen et al., 2006; Berton et al., 2009; Feyder et al., 2016). Thus, it appears that disruption of the cAMP/PKA/ERK1/2/ΔFosB cascade at numerous levels has the potential to delay or prevent the development of dyskinesia.

While D1R-linked cAMP/PKA signaling has been associated with dyskinesiogenic effects of L-DOPA, some evidence suggests that recruitment of β-arrestin to the D1R may have anti-dyskinetic effects, while also promoting motor improvement in animal models (Urs et al., 2015; Zhang et al., 2019). For example, in β-arrestin-2 KO mice, L-DOPA-induced forward locomotion was reduced, while dyskinesia was potentiated (Urs et al., 2015). However, it remains difficult to reconcile the apparent importance of β-arrestin with the efficacy of G protein-biased D1/D5R agonists (Gray et al., 2018; Riesenberg et al., 2020; Young et al., 2020). Most notably, in a recent pre-clinical evaluation of the G protein-biased partial agonist Tavapadon in a non-human primate model of PD, this drug alleviated motor symptoms while producing less dyskinesia than L-DOPA (Young et al., 2020). Thus, it is clear that further research is required to understand the role of specific signaling pathways in the therapeutic and adverse effects of ligands targeting the D1R.

### D1/D5R Agonists as Therapeutics for Cognitive Impairment

#### Cognitive Impairment and the Role of Dopamine

The D1R is an important regulator of cognitive functions, including spatial learning, working memory, executive function, and visuospatial functions (reviewed in Cools, 2006; Arnsten et al., 2015). The greatest risk factor for cognitive impairment is aging and advanced age in humans is also associated with decreases in cortical dopamine activity (Volkow et al., 1998) and a decline in D1R density in both the frontal cortex (de Keyser et al., 1990) and striatum (Suhara et al., 1991). Findings such as these argue for the potential of D1R-targeted therapies for ameliorating age-related cognitive decline. Aside from natural aging, multiple disease states are also associated with deficits in cognition including mood disorders, neurodegenerative diseases like PD, and schizophrenia. Presently there are very limited options for pharmacological management of cognitive impairment, whether associated with age or any of these conditions (Cools, 2006; Arnsten et al., 2015).

Despite endeavors to develop D1/D5R-targeted therapies to counteract cognitive decline, several challenges have so far prevented the realization of clinically viable D1R-targeting therapies. In the following sections, we will present animal and human studies that have investigated the role of the D1R in regulating various domains of cognitive function, and we will discuss efforts toward the clinical development of D1/D5R agonists for cognitive improvement.

#### D1R Pharmacodynamics in Cognition: The Prefrontal Cortex and Inverted U Dose-Relationship

The D1R is enriched in the prefrontal cortex (PFC), including in humans and monkeys, where it has been found to be localized to dendritic shafts, dendritic spines and axon terminals (Smiley et al., 1994; Paspalas and Goldman-Rakic, 2005; Bordelon-Glausier et al., 2008). The D5R is also abundant in the PFC (Meador-Woodruff et al., 1992; Ciliax et al., 2000) and in non-human primates has been shown to be co-expressed in a subset of D1R expressing neurons (Bergson et al., 1995; Bordelon-Glausier et al., 2008). While PFC function is implicated in many cognitive processes, prefrontal D1R signaling has been most extensively studied for its ability to regulate working memory, a cognitive process roughly defined as the ability to generate and update mental representations of information that can be used to guide subsequent actions (Arnsten et al., 2015). Early evidence that D1/D5R signaling in the PFC is an important driver of working memory came from observations that D1/D5R antagonists administered directly into the PFC impaired performance on working memory-dependent tasks in non-human primates (Sawaguchi and Goldman-Rakic, 1991, 1994). Perhaps unexpectedly, D1/D5R *agonist* infusion into the PFC also impaired working memory (Zahrt et al., 1997; Gamo et al., 2015). Subsequent studies have described an “inverted-U” dose response curve for PFC D1/D5R activation, in which the maximum benefit on working memory is obtained at intermediate doses, with the highest doses even impairing cognition (Zahrt et al., 1997; Granon et al., 2000; Vijayraghavan et al., 2007; Roberts et al., 2010).

At the circuit level, the effect of D1R signaling in the PFC on working memory has been proposed to occur through the regulation of so called “delay cells” (Arnsten et al., 2015). Delay cells are PFC neurons whose activity is thought to “hold” specific sensory information as a mental representation during the delay between a sensory stimulus and subsequent action. Direct iontophoretic application of D1/D5R ligands affects delay cell firing with an inverted-U dose response in monkeys performing delay-dependent working memory tasks (Williams and Goldman-Rakic, 1995; Vijayraghavan et al., 2007; Yang et al., 2021). In effect, moderate doses of D1/D5R agonist are thought to suppress “background” activity of delay cells, increasing the signal-to-noise sensitivity (Vijayraghavan et al., 2007; Yang et al., 2021). In contrast high doses of either D1/D5R agonists or antagonists can inhibit the firing of delay cells (Williams and Goldman-Rakic, 1995; Vijayraghavan et al., 2007). It is further proposed that moderate levels of D1/D5R stimulation can have excitatory effects on delay cell firing which contribute to the working memory benefits of D1/D5R agonism (Henze et al., 2000; Wang et al., 2019a). In line this model, the non-catecholamine agonist PF-3628 (described as a low affinity G protein-biased agonist), was found to excite delay cell firing in aged non-human primates engaged in a working memory task (Wang et al., 2019a). This effect was lost at high concentrations of the agonist and contrasted with the effect of a typical high-affinity balanced agonist, SKF 81297, which was found to suppress delay cell firing even at low concentrations (Wang et al., 2019a). When a related non-catechol agonist, PF-6142 (a G protein-biased partial agonist) was evaluated across a range of behavioral measures, it did not produce the expected inverted-U dose response pattern within the dose range tested (Kozak et al., 2020). These findings, obtained with two pharmacologically divergent D1 agonists, strongly suggest that in order to be clinically viable, D1/D5R agonists designed to treat cognitive impairment will need to avoid over-stimulating D1/D5R signaling.

#### D1R Signaling in Cognition: Beyond the Prefrontal Cortex

While clinical interest in targeting the D1R to treat cognitive impairment has focused on cognitive processes such as working memory, predominantly thought to depend on the PFC, D1R signaling in other brain regions also contributes to aspects of cognitive function. For example, in the striatum, D1R signaling plays a well-established role in reward-associated learning (reviewed in Cox and Witten, 2019). The role of dopamine in striatal reinforcement learning is described by the reward-prediction-error model (reviewed in Watabe-Uchida et al., 2017) in which bursts of striatal dopamine encode discrepancies between predicted vs. received sizes of rewards. The bursts of striatal dopamine release that result from unexpected rewards encode positive-reinforcement learning (Watabe-Uchida et al., 2017), and this appears to rely on D1R signaling (Schultz, 2016). Whether D1Rs also play a role in negative-reinforcement associative learning remains controversial (Nakanishi et al., 2014; Soares-Cunha et al., 2016; Higa et al., 2017).

In the hippocampus, genetic, and pharmacological evidence suggests that D1Rs play an important role in regulating long-term potentiation and specific forms of learning and memory (Huang and Kandel, 1995; Smith et al., 1998; El-Ghundi et al., 1999; Li et al., 2003; Hansen and Manahan-Vaughan, 2014). Although both D1R and D5R are present in the hippocampus, targeted deletion of the D1R but not D5R results in deficits in hippocampal LTP (Granado et al., 2008; Ortiz et al., 2010), contextual fear conditioning (Ortiz et al., 2010; Sariñana et al., 2014) and spatial learning (Granado et al., 2008; Ortiz et al., 2010; Sariñana and Tonegawa, 2016), therefore suggesting a predominant role for the D1R in these processes.

In the amygdala, the D1R has also been implicated in a number of cognition-related effects. In adult rats, these receptors were reported to play a role, for example, in the acquisition of a sucrose-rewarded lever-pressing task (Andrzejewski et al., 2005), in the acquisition of contextual fear conditioning (Heath et al., 2015), in memory consolidation in an object recognition task (Rossato et al., 2013), and in attentional performance in the 5-choice visual detection choice task (Smith et al., 2015). However, a limitation of these studies is that they all used intracerebral injection of the D1/D5R antagonist SCH 23390, a drug which also possesses activity at 5-HT2 receptors (Hyttel, 1983; Briggs et al., 1991).

#### Task- and Baseline-Dependent Variability in the Effect of D1R Agonists on Cognitive Function

In evaluating the effects of D1/D5R agonism on cognitive performance it appears that differences in specific measures of cognition, and differences in the baseline cognitive performance of subjects, are both important factors affecting the drug response. For example, in one study the D1/D5R partial agonist SKF38393 was found to improve attentional performance and decision making, but only in rats with low baseline performance in these tasks (Granon et al., 2000). Similarly, greater improvements in spatial working memory were found following a regimen of D1/D5R agonist in memory-impaired, older rhesus monkeys compared to young, unimpaired monkeys (Castner and Goldman-Rakic, 2004). To complicate matters further, D1R activation may exert divergent effects on different cognitive functions. In one study, for example, the D1/D5R agonist SKF 38393 was tested in rats that had been acutely pretreated with scopolamine in order to impair performance in several learning-related tasks; SKF 38393 either counteracted or exacerbated these deficits, in a task-dependent fashion (Amico et al., 2007). In another study, two D1/D5R agonists were found to have no effect on spatial working memory in non-human primates when given alone, but both drugs ameliorated working memory deficits induced by ketamine (Roberts et al., 2010).

#### D1R-Dependent Signaling Pathways Associated With Cognitive Improvement

Little is currently known about the specific D1R-mediated intracellular signaling pathways that regulate cognition in the PFC. The inhibitory effect of high concentrations of D1/D5R agonists on delay cell firing has been proposed to occur via cAMP-dependent signaling to HCNs (hyperpolarization-activated cyclic nucleotide-gated ion channels) (Vijayraghavan et al., 2007; Paspalas et al., 2013; Gamo et al., 2015). Some evidence also suggests that cognitive improvements are related to the ability of D1R signaling to regulate synaptic plasticity through controlling the expression and surface tracking of NMDA glutamate receptors (Gurden et al., 2000; Baldwin et al., 2002; Gao and Wolf, 2008). NMDA receptor regulation is mediated in part by PKA (Gurden et al., 2000; Baldwin et al., 2002) but in cultured cortical neurons has also been found to depend on Fyn kinases (Hu et al., 2010). Attributing behavioral or circuit-level effects to specific D1R-mediated signaling pathways is further complicated by the fact that D1R receptors are expressed in, and may differentially regulate, multiple cell types in the PFC (Anastasiades et al., 2019). For example, the D1R that is expressed in mouse PFC layers 5 and 6 has excitatory effects on local pyramidal neurons as well as certain types of interneuron (Anastasiades et al., 2019). However, the D1R has also been found in a subset of presynaptic glutamatergic terminals in the PFC, where it serves an inhibitory function, suppressing vesicular release through a PKA dependent mechanism (Burke et al., 2018). Dissecting the relationship between specific signaling processes and cognitive outcomes is further complicated by the fact that the D5R is also expressed throughout the PFC (Bergson et al., 1995; Bordelon-Glausier et al., 2008), and appears to regulate signaling pathways distinct from those of the D1R (Perreault et al., 2013), actions which could also affect cognitive performance (Carr et al., 2017).

From a therapeutic perspective, an important unaddressed question is whether biased D1/D5R agonists could provide an improved therapeutic profile compared to traditional balanced ligands. One potential advantage of G protein biased D1R agonists is that they have been found to preclude desensitization of the receptor (Gray et al., 2018), which could allow for repeated dosing without the rapid development of tolerance which has been observed with balanced agonists. A recent study compared two D1/D5R agonists that induced high vs. low levels of β-arrestin recruitment, respectively, but had equal efficacy with respect to cAMP signaling. Both drugs depressed PFC neuronal firing rate during a working memory task, and the drug with full β-arrestin activity produced greater improvements in task performance (Yang et al., 2021). This study represents the first attempt to explicitly examine the role of D1R-mediated G protein vs. arrestin signaling in cognition, and although preliminary, suggests a role for β-arrestin signaling in the pro-cognitive effects of D1R agonists. However, it should be noted that this finding could contradict the previously described positive findings pertaining to G protein-biased agonists with either full (Wang et al., 2019a) or partial (Kozak et al., 2020) efficacy on cAMP signaling. To determine the optimal D1/D5R pharmacology for treating cognitive dysfunction more systematic comparisons will be required to further elucidate the relationship between specific signaling pathways and cognitive function.

#### Pro-cognitive Effects of D1/D5R Agonists in Human Subjects

The effects of D1/D5R stimulation on human cognition are complex, and as illustrated in the following paragraphs, appear to recapitulate several features identified in animal studies, including an inverted-U dose-response relationship, and a dependence on baseline state. In healthy subjects, D1R density in the PFC was found to have an inverted-U relationship with working memory performance (Takahashi et al., 2008), reinforcing the concept that over- or under-stimulation of D1Rs could negatively affect cognition. An early indication that D1R stimulation might promote cognition in human subjects came from studies using the non-selective D1/D2 agonist pergolide (Müller et al., 1998). When compared to a D2-selective agonist, pergolide produced greater working memory improvements in a visuospatial delayed response task (Müller et al., 1998) suggesting a role for D1R in human working memory. However, not all pergolide investigations in human subjects have produced convergent findings; one study detected no effect (Bartholomeusz et al., 2003) while another revealed positive effects of pergolide only in subjects possessing high working memory capacity—a finding which in turn diverges from the baseline-dependent effect of D1/D5R agonists reported elsewhere (Kimberg and D'Esposito, 2003).

Pergolide is also used in the treatment of Parkinson's Disease, and its effect on cognition in this context once again appear to be variable, with some studies reporting a positive effect on working memory (Costa et al., 2009) but others finding no effect on cognition (Brusa et al., 2005). However, in PD the effects on cognition of the degeneration of nigral dopamine neurons, the resultant dopamine depletion in both striatal and extra-striatal regions and subsequent dopaminergic therapy are complex and multi-factorial (reviewed in Cools, 2006). A systematic review of possible cognitive effects of dopaminergic therapies in PD concluded that therapeutic effects, where reported, were variable and that more investigation would be required in order to comprehensively evaluate the effect of therapies, both individually and in comparison to other treatments (Poletti and Bonuccelli, 2013). Of particular note, very few studies have evaluated the *long-term* effects of dopaminergic treatment on cognitive function in PD. Most clinical studies of dopaminergic therapy and cognition in PD have evaluated the effects of either D2-selective, or D1/D2 non-selective dopamine agonists. However, trials are now in progress that will evaluate the effect of selective D1/D5R agonists in PD patients, potentially shedding more light on the role of D1-class receptors in cognitive performance in this disease.

Disruptions in D1R-mediated dopaminergic signaling in the PFC appear to contribute to the cognitive symptoms of schizophrenia (reviewed in Arnsten et al., 2017). Several clinical studies have now evaluated the effects of the D1 agonist dihydrexidine in patients with schizophrenia or schizotypal personality disorder (George et al., 2007; Mu et al., 2007; Rosell et al., 2015; Girgis et al., 2016). Overall, these studies have produced limited, but promising findings, including increased PFC perfusion and some improvements in cognitive function. However, as in Parkinson's Disease (Blanchet et al., 1998), the poor oral bioavailability and rapid metabolism of the drug are acknowledged to be major limiting factors that preclude a comprehensive assessment of dihydrexidine.

Recently, attention has shifted to non-catechol D1R ligands as potential therapeutics for cognitive impairment. As described previously, these drugs have improved pharmacokinetic properties compared to previous generations of D1/D5R agonists. Preclinical assessment of the G protein-biased partial D1/D5R agonist CVL-751 (Tavapadon) improved measures of cognition and working memory in rodents and non-human primates (Kozak et al., 2020). However, initial clinical evaluations of a related compound, PF-06412562, either in healthy volunteers with low working memory capacity, or in patients with stable schizophrenia, have not shown significant improvement compared to placebo (Arce et al., 2019; Balice-Gordon et al., 2020). PF-06412562 also appears to affect other aspects of cognitive function. In particular, improvements have been reported in cost-benefit decision making (Soutschek et al., 2020a), and in the flexible processing of Pavlovian cues that predict reward outcomes (Soutschek et al., 2020b).

#### Positive Allosteric Modulators for Treatment of Cognitive Impairment

Positive allosteric modulators (PAMs) offer an alternative strategy to D1R agonists for the treatment of impaired cognition (Svensson et al., 2019). Since D1R-targeted PAMs enhance the actions of endogenous dopamine without directly activating the receptor, they offer some potential advantages (Bruns et al., 2018; Meltzer et al., 2019). First, PAMs would be expected to preserve temporal coding of released transmitter, whereas receptor agonists would tend to override it. Second, high doses of PAMs, unlike receptor agonists, produce less receptor desensitization. Consequently, D1R PAMs are not subject to the inverted-U dose response relationship that limits the therapeutic window for D1R agonists (Svensson et al., 2017; Bruns et al., 2018; Meltzer et al., 2019). To date, a D1R PAM has been found to exert pro-cognitive effects in mice expressing the human D1R (Meltzer et al., 2019), and clinical evaluation has recently begun for the D1R PAM LY3154207 (Mevidalen). Initial reports suggest that this drug is safe and well tolerated (Wilbraham et al., 2021a), supporting continued clinical testing. In Lewy body dementia, a disease characterized by both Parkinsonism and dementia or cognitive impairment, Mevidalen did not significantly improve cognition, but did improve motor function when administered alongside standard dopaminergic therapies (Biglan et al., 2021). In this exciting new avenue of drug development, it will be important to try to relate pro-cognitive drug effects to specific intracellular signaling pathways downstream of the D1 receptor.

## Summary and Looking Ahead

After four decades of investigation, it is only in the last few years that we have seen the breakthroughs in medicinal chemistry that might allow for D1R-targeted therapeutics to reach clinical deployment. Although our understanding of D1R signaling is far from complete, these decades of research have left us with an extensive base of pharmacological knowledge with which to guide future development of D1R-targeted therapeutics that may ultimately improve the lives of patients.

## Data Availability Statement

The original contributions presented in the study are included in the article/supplementary material, further inquiries can be directed to the corresponding author/s.

## Author Contributions

This review was written as a first draft by JJ-T, HM, and EP. PC and TH reviewed and edited it. All authors contributed to the article and approved the submitted version.

## Funding

This work was supported by grants from the Weston Brain Institute and Canadian Institutes of Health Research (PJT-174985). JJ-T was supported by doctoral studentships from the Canadian Institutes of Health Research and the McGill Healthy Brains for Healthy Lives initiative. TH was holder of the Canadian Pacific Chair in Biotechnology.

## Conflict of Interest

The authors declare that the research was conducted in the absence of any commercial or financial relationships that could be construed as a potential conflict of interest.

## Publisher's Note

All claims expressed in this article are solely those of the authors and do not necessarily represent those of their affiliated organizations, or those of the publisher, the editors and the reviewers. Any product that may be evaluated in this article, or claim that may be made by its manufacturer, is not guaranteed or endorsed by the publisher.
